# Supplementary material for: Clinical Value of Circulating miRNA in Diagnosis, Prognosis, Screening and Monitoring Therapy of Pancreatic Ductal Adenocarcinoma—A Review of the Literature
Source: Int J Mol Sci. 2023 Mar 7;24(6):5113. doi: 10.3390/ijms24065113 (PMC10049684; doi:10.3390/ijms24065113)
Supplement: Supplementary file 1 [file ijms-24-05113-s001.zip › ijms-2239869-supplementary.pdf]

Supplementary Table S1. Characteristics of classic diagnostic methods of pancreatic ductal adenocarcinoma. [1]

Diagnostic tool	Advantages	Disadvantages
Transabdominal ultrasound	• non-invasive<br>• relatively cost-effective	• difficulties in imaging of pancreatic body and tail cancers<br>• highly dependent on the operator's experience
Computed tomography	• non-invasive<br>• good spatial resolution<br>• good temporal resolution<br>• wide anatomic coverage<br>• best performance for the evaluation of vascular involvement	• may not depict small metastases to the liver or peritoneum
EUS-guided fine-needle aspiration	• high-resolution images of the pancreas<br>• ability to obtain specimens for histopathological diagnosis	• an invasive procedure, however considered safe and accurate
Magnetic resonance imaging	• non-invasive<br>• greater soft-tissue contrast of MRI compared with that of CT	• worse spatial resolution compared to CT (requires relatively more time to perform the imaging)

Supplementary Table S2. Diagnostic performance of circulating miRNA (CS – clinical stage, PC-pancreatic cancer, CP-chronic pancreatitis, IPMN – intraductal papillary mucinous neoplasm AUC – area under the curve), 95% CI are provided in square brackets.

*-analysis with Abcam Fireplex™ platform

† - **provided with miRBase: the microRNA database**

miRNA	Location and predicted target genes †	Author	Year	Normalization control	Source of sample	Expression level compared to non-cancer controls	CS of PC patients	Control	AUC	Sensitivity	Specificity
miR-21	chr17:	Abue et	2015	exogenous cel-	plasma	upregulated	I-IV	healthy patients	0.736	-	-

	59841266-59841337 [+] [82] [83]	al.[84]		miR-39, endogenous miR-16				and IPMN			
miR-483-3p	chr11: 2134134-2134209 [-] [137]	Abue et al.[84]	2015	exogenous cel-miR-39, endogenous miR-16	plasma	upregulated	I-IV	healthy patients and IPMN	0.740	-	-
miR-1202	chr 6: 155946797-155946879 [15]	Aita et al. [16]	2021	exogenous UniSp6, exogenous spike-in mix (UniSp2, UniSp4, UniSp5)	serum	downregulated	I-III	healthy patients	0.8833 [0.7246-1.00]	-	-
miR-1275	chr 6: 33999972-34000051 [-] [25]	Aita et al. [16]	2021	exogenous UniSp6, exogenous spike-in mix (UniSp2, UniSp4, UniSp5)	serum	downregulated	I-III	healthy patients	0.8333 [0.60049-1.00]	-	-
miR-1915-3p	chr10: 21496562-21496641 [-] [59]	Aita et al. [16]	2021	exogenous UniSp6, exogenous spike-in mix (UniSp2, UniSp4, UniSp5)	serum	upregulated	I-III	healthy patients	0.8833 [0.70258-1.00]	-	-
miR-2392	chr14: 100814491-100814574 [+] [105]	Aita et al. [16]	2021	exogenous UniSp6, exogenous spike-in mix (UniSp2, UniSp4, UniSp5)	serum	downregulated	I-III	healthy patients	0.8667 [0.75085-0.98249]	-	-
miR-3135-b	chr6: 32749912-	Aita et al. [16]	2021	exogenous UniSp6,	serum	upregulated	I-III	healthy patients	0.8333 [0.70987-0.95680]	-	-

	32749979 [-] [115]			exogenous spike-in mix (UniSp2, UniSp4, UniSp5)							
miR-3679-5p	chr2: 134127125-134127192 [+] [121]	Aita et al. [16]	2021	exogenous UniSp6, exogenous spike-in mix (UniSp2, UniSp4, UniSp5)	serum	downregulated	I-III	healthy patients	0.85 [0.6431-1.00]	-	-
miR-371b-5p	chr19: 53787677-53787742 [-] [122]	Aita et al. [16]	2021	exogenous UniSp6, exogenous spike-in mix (UniSp2, UniSp4, UniSp5)	serum	upregulated	I-III	healthy patients	0.9333 [0.84430-1.00]	-	-
miR-4327	chr21: 30375294-30375378 [-] [130]	Aita et al. [16]	2021	exogenous UniSp6, exogenous spike-in mix (UniSp2, UniSp4, UniSp5)	serum	upregulated	I-III	healthy patients	0.9333 [0.84430-1.00]	-	-
miR-4466	chr6: 156779678-156779731 [-][131]	Aita et al. [16]	2021	exogenous UniSp6, exogenous spike-in mix (UniSp2, UniSp4, UniSp5)	serum	upregulated	I-III	healthy patients	0.8500 [0.58529-1.00]	-	-
miR-4516	chr16: 2133119-2133204 [+] [132]	Aita et al. [16]	2021	exogenous UniSp6, exogenous spike-in mix	serum	upregulated	I-III	healthy patients	0.9167 [0.78255-1.00]	-	-

				(UniSp2, UniSp4, UniSp5)							
miR-4655-3p	chr7: 1844180-1844253 [-] [134]	Aita et al. [16]	2021	exogenous UniSp6, exogenous spike-in mix (UniSp2, UniSp4, UniSp5)	serum	upregulated	I-III	healthy patients	0.8667 [0.75085-0.98249]	-	-
miR-4669	chr9: 134379411-134379472 [+] [135]	Aita et al. [16]	2021	exogenous UniSp6, exogenous spike-in mix (UniSp2, UniSp4, UniSp5)	serum	downregulated	I-III	healthy patients	0.8333 [0.56959-1.00]	-	-
miR-4687-3p	chr11: 3856062-3856141 [+] [136]	Aita et al. [16]	2021	exogenous UniSp6, exogenous spike-in mix (UniSp2, UniSp4, UniSp5)	serum	upregulated	I-III	healthy patients	0.8833 [0.70258-1.00]	-	-
miR-5100	chr10: 42997563-42997681 [+] [141]	Aita et al. [16]	2021	exogenous UniSp6, exogenous spike-in mix (UniSp2, UniSp4, UniSp5)	serum	upregulated	I-III	healthy patients	0.8667 [0.67646-1.00]	-	-
miR-574-3p	chr4: 38868032-38868127 [+] [142]	Aita et al. [16]	2021	exogenous UniSp6, exogenous spike-in mix (UniSp2, UniSp4,	serum	downregulated	I-III	healthy patients	0.8333 [0.59329-1.00]	-	-

				UniSp5)							
miR-6089	chrX: 2609191-2609254 [+] [145]	Aita et al. [16]	2021	exogenous UniSp6, exogenous spike-in mix (UniSp2, UniSp4, UniSp5)	serum	upregulated	I-III	healthy patients	0.8667 [0.63230-1.00]	-	-
miR-6125	chr12: 62260359-62260454 [+] [146]	Aita et al. [16]	2021	exogenous UniSp6, exogenous spike-in mix (UniSp2, UniSp4, UniSp5)	serum	upregulated	I-III	healthy patients	0.8833 [0.67896-1.00]	-	-
miR-6126	chr16: 3485381-3485469 [-] [147]	Aita et al. [16]	2021	exogenous UniSp6, exogenous spike-in mix (UniSp2, UniSp4, UniSp5)	serum	upregulated	I-III	healthy patients	0.90 [0.79524-1.00]	-	-
miR-6749-5p	chr11: 64902387-64902455 [-] [154]	Aita et al. [16]	2021	exogenous UniSp6, exogenous spike-in mix (UniSp2, UniSp4, UniSp5)	serum	downregulated	I-III	healthy patients	0.8667 [0.0858-0.69858]	-	-
miR-6800-5p	chr19: 49832018-49832099 [+] [155]	Aita et al. [16]	2021	exogenous UniSp6, exogenous spike-in mix (UniSp2, UniSp4, UniSp5)	serum	upregulated	I-III	healthy patients	0.90 [0.74399-1.00]	-	-
miR-6821-5p	chr22:	Aita et al.	2021	exogenous	serum	upregulated	I-III	healthy patients	0.9667 [0.88761-	-	-

	49962866-49962939 [+] [156]	[16]		UniSp6, exogenous spike-in mix (UniSp2, UniSp4, UniSp5)					1.00]		
miR-6850-5p	chr8: 144791931-144791991 [-] [157]	Aita et al. [16]	2021	exogenous UniSp6, exogenous spike-in mix (UniSp2, UniSp4, UniSp5)	serum	upregulated	I-III	healthy patients	0.9167 [0.77041-1.00]	-	-
miR-6869-5p	chr20: 1392900-1392961 [-] [158]	Aita et al. [16]	2021	exogenous UniSp6, exogenous spike-in mix (UniSp2, UniSp4, UniSp5)	serum	upregulated	I-III	healthy patients	0.9167 [0.74206-1.00]	-	-
miR-7107-5p	chr12: 121444273-121444352 [-] [161]	Aita et al. [16]	2021	exogenous UniSp6, exogenous spike-in mix (UniSp2, UniSp4, UniSp5)	serum	downregulated	I-III	healthy patients	0.8333 [0.59329-1.00]	-	-
miR-7110-5p	chr3: 123161794-123161879 [+] [162]	Aita et al. [16]	2021	exogenous UniSp6, exogenous spike-in mix (UniSp2, UniSp4, UniSp5)	serum	downregulated	I-III	healthy patients	0.8167 [0.55014-1.00]	-	-
miR-8485	chr2: 50696172-50696262 [-]	Aita et al. [16]	2021	exogenous UniSp6, exogenous	serum	downregulated	I-III	healthy patients	0.8833 [0.67896-1.00]	-	-

	[167]			spike-in mix (UniSp2, UniSp4, UniSp5)							
miR-939-5p	chr8: 144394149-144394230 [-] [171]	Aita et al. [16]	2021	exogenous UniSp6, exogenous spike-in mix (UniSp2, UniSp4, UniSp5)	serum	upregulated	I-III	healthy patients	0.90 [0.79524-1.00]	-	-
miR-181d	chr19: 13874875-13875011 [+] [50][51]	Akamatsu et al. [52]	2016	exogenous cel-miR-39	serum	upregulated	I-IV	autoimmune pancreatitis	0.882 [0.813 - 0.952]	81%	80%
miR-193b	chr16: 14303967-14304049 [+] [61] [62]	Akamatsu et al. [52]	2016	exogenous cel-miR-39	serum	upregulated	I-IV	autoimmune pancreatitis	0.804 [0.718 - 0.892]	79%	73%
miR-34a	chr1: 9151668-9151777 [-] [119][120]	Akamatsu et al. [52]	2016	exogenous cel-miR-39	serum	upregulated	I-IV	autoimmune pancreatitis	0.844 [0.754 - 0.935]	81%	80%
miR-7	chr9: 83969748-83969857 [-] [159] [160]	Akamatsu et al. [52]	2016	exogenous cel-miR-39	serum	upregulated	I-IV	autoimmune pancreatitis	0.723 [0.603 - 0.844]	72%	73%
miR-21	chr17: 59841266-59841337 [+] [82] [83]	Alemar et al. [85]	2016	exogenous cel-miR-39	serum	upregulated	I-IV	healthy patients	0.889 [0.76–1.00	82.6%	77.8%
miR-34a	chr1: 9151668-9151777 [-] [119][120]	Alemar et al. [85]	2016	exogenous cel-miR-39	serum	upregulated	I-IV	healthy patients	0.865 [0.71–1.00]	91.3%	77.8%
miR-196b	chr7:	Bartsch et	2018	endogenous	serum	upregulated	I-III	healthy patients	0.8938 [0.7395-	80%	50%

	27169480-27169563 [-] [66][67]	al. [68]		miR-24					1.048]		
miR-182	chr7: 129770383-129770492 [-] [53] [54]	Chen et al. [55]	2014	endogenous RNU6B	plasma	upregulated	I-IV	healthy patients, CP	0.775 [0.721–0.856]	64.1 %	82.6 %
miR -106b	chr 7: 100093993-100094074 [6]	Cote et al. [7]	2014	endogenous miRNA-425-5p	plasma	upregulated	I-IV	healthy patients, CP	0.999	100%	98%
miR-10b	chr 2: 176150303-176150412 [13][14]	Cote et al. [7]	2014	endogenous miRNA-425-5p	plasma	upregulated	I-IV	healthy patients, CP	0.980	95%	100%
miR-155	chr21: 25573980-25574044 [+] [34][35]	Cote et al. [7]	2014	endogenous miRNA-425-5p	plasma	upregulated	I-IV	healthy patients, CP	0.975	93%	100%
miR-212	chr17: 2050271-2050380 [-] [89] [90]	Cote et al. [7]	2014	endogenous miRNA-425-5p	plasma	upregulated	I-IV	healthy patients, CP	0.900	90%	83%
miR-30c	chr1: 40757284-40757372 [+] [113][114]	Cote et al. [7]	2014	endogenous miRNA-425-5p	plasma	upregulated	I-IV	healthy patients, CP	0.938	73%	96%
miR-25	chr7: 100093560-100093643 [-] [107] [108]	Deng et al.[109]	2016	directly normalized to the serum volume	serum	upregulated	I-IV	healthy patients, CP	0.915 [0.893-0.937]	-	-
miR-130a-3p*	chr11: 57641198-57641286	Dittmar et al. [29]	2021	endogenous miR-17–5p, miR-20a-5p,	plasma	upregulated	II	healthy patients	0.74 [0.63-0.84]	-	-

	[+][28]			miR-93–5p							
miR-222–3p*	chrX: 45747015-45747124 [-] [97]	Dittmar et al. [29]	2021	endogenous miR-17–5p, miR-20a-5p, miR-93–5p	plasma	upregulated	II	healthy patients	0.70 [0.58-0.81]	-	-
miR-34a-5p*	chr1: 9151668-9151777 [-] [119][120]	Dittmar et al. [29]	2021	endogenous miR-17–5p, miR-20a-5p, miR-93–5p	plasma	upregulated	II	healthy patients	0.77 [0.66- 0.87]	-	-
miR-642		Fathi et al. [151]	2022	endogenous RNU6B	plasma	upregulated	-	patients with gastrointestinal symptoms, without malignancy	0.63	58%	78%
miR-8073	chr13: 110340958-110341029 [+] [166]	Fathi et al. [151]	2022	endogenous RNU6B	plasma	upregulated	-	patients with gastrointestinal symptoms, without malignancy	0.82	77%	78%
let-7d-3p	chr 9: 94178834-94178920 [4]	Franklin et al [5]	2018	endogenous mean expression value normalization	plasma	upregulated	I-II	healthy patients	0.75 [0.61-0.89]	-	-
miR-101–3p	chr 1: 65058434-65058508 [10]	Franklin et al [5]	2018	endogenous mean expression value normalization	plasma	downregulated	I-II	healthy patients	0.75 [0.60-0.89]	-	-
miR-106b-5p	chr 7: 100093993-100094074 [6]	Franklin et al [5]	2018	endogenous mean expression value normalization	plasma	downregulated	I-II	healthy patients	0.81 [0.68-0.94]	-	-
miR-122–5p	chr 18: 58451074-	Franklin et al [5]	2018	endogenous mean	plasma	upregulated	I-II	healthy patients	0.75 [0.60-0.89]	-	-

	58451158 [17]			expression value normalization							
miR-130b-3p	chr11: 57641198-57641286 [+][28]	Franklin et al [5]	2018	endogenous mean expression value normalization	plasma	upregulated	I-II	healthy patients	0.80 [0.67-0.92]	-	-
miR-144–3p	chr17: 28861533-28861618 [-] [33]	Franklin et al [5]	2018	endogenous mean expression value normalization	plasma	downregulated	I-II	healthy patients	0.81 [0.68-0.94]	-	-
miR-197–3p	chr1: 109598893-109598967 [+] [69]	Franklin et al [5]	2018	endogenous mean expression value normalization	plasma	upregulated	I-II	healthy patients	0.76 [0.62-0.90]	-	-
miR-22–5p	chr17: 1713903-1713987 [-] [104]	Franklin et al [5]	2018	endogenous mean expression value normalization	plasma	upregulated	I-II	healthy patients	0.78 [0.64-0.92]	-	-
miR-24–3p	chr9: 95086021-95086088 [+] [106]	Franklin et al [5]	2018	endogenous mean expression value normalization	plasma	upregulated	I-II	healthy patients	0.78 [0.65-0.92]	-	-
miR-26a-5p	chr3: 37969404-37969480 [+] [111]	Franklin et al [5]	2018	endogenous mean expression value normalization	plasma	downregulated	I-II	healthy patients	0.77 [0.62-0.91]	-	-
miR-34a-5p	chr1: 9151668-9151777 [-]	Franklin et al [5]	2018	endogenous mean expression	plasma	upregulated	I-II	healthy patients	0.82 [0.69-0.94]	-	-

	[119][120]			value normalization							
miR-423–3p	chr17: 30117079-30117172 [+] [127]	Franklin et al [5]	2018	endogenous mean expression value normalization	plasma	upregulated	I-II	healthy patients	0.76 [0.62-0.90]	-	-
miR-451a	chr17: 28861369-28861440 [-] [133]	Franklin et al [5]	2018	endogenous mean expression value normalization	plasma	downregulated	I-II	healthy patients	0.77 [0.63-0.91]	-	-
miR-574–3p	chr4: 38868032-38868127 [+] [142]	Franklin et al [5]	2018	endogenous mean expression value normalization	plasma	upregulated	I-II	healthy patients	0.83[0.70-0.95]	-	-
miR-885–5p	chr3: 10394489-10394562 [-] [168]	Franklin et al [5]	2018	endogenous mean expression value normalization	plasma	upregulated	I-II	healthy patients	0.79 [0.66-0.93]	-	-
miR-22-3p	chr17: 1713903-1713987 [-] [101]	Ganepola et al. [102]	2014	endogenous miR-3196	plasma	upregulated	IIA-IIB	healthy patients	0.86 [0.70-1.00]	-	-
miR-642b-3p	chr19: 45674932-45675008 [-] [152]	Ganepola et al. [102]	2014	endogenous miR-3196	plasma	upregulated	IIA-IIB	healthy patients	0.79 [0.59-0.98]	-	-
miR-885-5p	chr3: 10394489-10394562 [-] [168]	Ganepola et al. [102]	2014	endogenous miR-3196	plasma	upregulated	IIA-IIB	healthy patients	0.84 [0.68-1.00]	-	-
miR-373	chr19: 53788705-	Hua et al.[125]	2017	endogenous RNU6B	serum	downregulated	I-IV	healthy patients	0.852	80.6%	84.3%

	53788773 [+] [123][124]										
miR-429	chr1: 1169005-1169087 [+] [128]	Huang et al.[129]	2022	endogenous RNU6B	serum	downregulated	I-IV	healthy patients	0.858 [0.727-0.989]	-	-
miR-22-3p	chr17: 1713903-1713987 [-] [101]	Hussein et al.[103]	2017	endogenous miR-3196	serum	upregulated	IB,IIB,IV	healthy patients	0.943	97.14%	93.33%
miR-642b-3p	chr19: 45674932-45675008 [-] [152]	Hussein et al.[103]	2017	endogenous miR-3196	serum	upregulated	IB,IIB,IV	healthy patients	1.00	100%	100%
miR-885-5p	chr3: 10394489-10394562 [-] [168]	Hussein et al.[103]	2017	endogenous miR-3196	serum	upregulated	IB,IIB,IV	healthy patients	1.00	100%	100%
miR-107	chr 10: 89592747-89592827 [11]	Imamura et al.[12]	2017	exogenous cel-miR-39	plasma	downregulated	I-IV	healthy patients	0.851	82%	68.8%
miR-607	chr10: 96828669-96828764 [-] [143]	Jiang et al. [144]	2021	endogenous RNU6	serum	downregulated	I-IV	healthy patients	0.785 [0.738–0.831]	64.7%	77.2%
miR-126-5p	chr 9: 136670602-136670686 [+][23]	Karasek et al. [24]	2018	endogenous miR-106a	plasma	upregulated	I-II	healthy patients	0.85	-	-
miR-1290	chr1: 18897071-18897148 [-] [26]	Karasek et al. [24]	2018	endogenous miR-106a	plasma	upregulated	I-II	healthy patients	0.78	-	-
miR-155	chr21:	Karasek et	2018	endogenous	plasma	upregulated	I-II	healthy patients	0.86	-	-

	25573980-25574044 [+] [34][35]	al. [24]		miR-106a							
miR-17-5p	chr13: 91350605-91350688 [+] [41]	Karasek et al. [24]	2018	endogenous miR-106a	plasma	upregulated	I-II	healthy patients	0.87	-	-
miR-21-5p	chr17: 59841266-59841337 [+] [82] [83]	Karasek et al. [24]	2018	endogenous miR-106a	plasma	upregulated	I-II	healthy patients	0.99	-	-
miR-375	chr2: 219001645-219001708 [-][126]	Karasek et al. [24]	2018	endogenous miR-106a	plasma	upregulated	I-II	healthy patients	0.73	-	-
miR-221	chrX: 45746157-45746266 [-] [94][95]	Kawaguchi et al.[96]	2013		plasma	upregulated	I-IV	healthy patients	0.743	-	-
miR-122-5p	chr 18: 58451074-58451158 [17]	Khan et al.[18]	2021	exogenous UniSp6	serum	upregulated	I-IV	healthy patients	0.988 [0.927–0.999]	98%	96%
miR-192-5p	chr11: 64891137-64891246 [-] [60]	Khan et al.[18]	2021	exogenous UniSp6	serum	upregulated	I-IV	healthy patients	0.720 [0.604–0.817]	74%	60%
miR-215-5p	chr1: 220117853-220117962 [-] [91]	Khan et al.[18]	2021	exogenous UniSp6	serum	upregulated	I-IV	healthy patients	0.832 [0.721–0.904]	72%	72%
miR-30b-5p	chr8: 134800520-134800607 [-] [112]	Khan et al.[18]	2021	exogenous UniSp6	serum	downregulated	I-IV	healthy patients	0.798 [0.691–0.883]	76%	68%
miR-320b	chr1:	Khan et	2021	exogenous	serum	upregulated	I-IV	healthy patients	0.922 [0.833–	84%	78%

	116671746-116671817 [+] [117]	al.[18]		UniSp6					0.970]		
miR-223	chrX: 66018870-66018979 [+] [98] [99]	Komatsu et al.[100]	2015	exogenous cel-miR-39	plasma	upregulated	I-II	healthy patients	0.8340	62%	94.1%
miR-200a	chr1: 1167863-1167952 [+] [72][73]	Li et al.[74]	2010	endogenous miR-16	serum	upregulated	-	healthy patients	0.861 [0.774–0.949]	84.4%	87.5%
miR-200b	chr1: 1167104-1167198 [+] [75] [76]	Li et al.[74]	2010	endogenous miR-16	serum	upregulated	-	healthy patients	0.85 [0.763–0.938]	71.1%	96.9%
miR-492	chr12: 94834398-94834513 [+] [139]	Lin et al.[140]	2014	exogenous cel-miR-39	serum	downregulated	I-IV	healthy patients	0.787 [0.689-0.885]	75.5%	70.0%
miR-663a	chr20: 26208186-26208278 [-] [153]	Lin et al.[140]	2014	exogenous cel-miR-39	serum	downregulated	I-IV	healthy patients	0.870 [0.793-0.948]	85.7%	80.0%
miR-196a	chr17: 48632490-48632559 [-] [64]	Liu et al. [46]	2020	endogenous RNU6B	plasma	upregulated	I-IV	healthy patients	0.865 [0.779-0.951]	72.5%	92.5%
miR-155	chr21: 25573980-25574044 [+] [34][35]	Liu et al. [36]	2012	exogenous cel-miR-39	plasma	upregulated	I-IV	healthy patients, CP	0.704 [0.646-0.762]	-	-
miR-16	chr13: 50048973-50049061 [-] [39] [40]	Liu et al. [36]	2012	exogenous cel-miR-39	plasma	upregulated	I-IV	healthy patients, CP	0.749 [0.694-0.803]	-	-
miR-181b	chr1:	Liu et al.	2012	exogenous cel-	plasma	upregulated	I-IV	healthy patients,	0.785 [0.735-	-	-

	198858873-198858982 [-] [44][45]	[36]		miR-39				CP	0.835]		
miR-196a	chr17: 48632490-48632559 [-] [64]	Liu et al. [36]	2012	exogenous cel-miR-39	plasma	upregulated	I-IV	healthy patients, CP	0.816 [0.768-0.864]	-	-
miR-21	chr17: 59841266-59841337 [+] [82] [83]	Liu et al. [36]	2012	exogenous cel-miR-39	plasma	upregulated	I-IV	healthy patients, CP	0.776 [0.725-0.827]	-	-
miR-210	chr11: 568089-568198 [-] [87][88]	Liu et al. [36]	2012	exogenous cel-miR-39	plasma	upregulated	I-IV	healthy patients, CP	0.755 [0.701-0.808]	-	-
miR-181b	chr1: 198858873-198858982 [-] [44][45]	Liu et al. [46]	2020	endogenous RNU6B	plasma	upregulated	I-IV	healthy patients	0.789 [0.681-0.898]	77.5%	85.0%
miR-210	chr11: 568089-568198 [-] [87][88]	Liu et al. [46]	2020	endogenous RNU6B	plasma	upregulated	I-IV	healthy patients	0.834 [0.745-0.923]	82.5%	80.0%
miR-181a	chr1: 198859044-198859153 [-] [42] [43]	Liu J. et al. [36]	2012	exogenous cel-miR-39	plasma	upregulated	I-IV	healthy patients, CP	0.774 [0.723-0.825]	-	-
miR-744	chr17: 12081899-12081996 [+] [163] [164]	Miyamae et al.[165]	2015	exogenous cel-miR-39	plasma	upregulated	I-IV	healthy patients	0.8307	59.3%	89.6%
miR-18a	chr13: 91350751-91350821 [+] [56] [57]	Morimura et al. [58]	2011		plasma	upregulated	I-IV	healthy patients	0.9369	-	-

miR-21-5p	chr17: 59841266-59841337 [+] [82]	Qu et al.[92]	2017	exogenous cel-miR-39	serum	upregulated	I-IV	healthy patients	0.78 [0.66–0.90]	77%	80%
miR-629	chr15: 70079372-70079468 [-] [148] [149]	Shi et al.[150]	2018		serum	upregulated	I-IV	healthy patients	0.765 [0.679-0.851]	-	-
miR-196		Škrha et al.[63]	2016	endogenous miR-191 and miR-454	serum	upregulated	-	healthy patients	0.74	-	-
miR-200		Škrha et al.[63]	2016	endogenous miR-191 and miR-454	serum	upregulated	-	healthy patients	0.79	-	-
miR-196b	chr7: 27169480-27169563 [-] [66][67]	Slater et al. [65]	2014	endogenous miR-24	serum	upregulated	I,II,IV	healthy patients	0.86	100%	78%
miR-196a	chr17: 48632490-48632559 [-] [64]	Slater et al. [65]	2014	endogenous miR-24	serum	upregulated	I,II,IV	healthy patients	0.97	90%	89%
miR-100	chr 11: 122152229-122152308 [8]	Stroese et al. [9]	2018	exogenous cel-miR-39	serum	upregulated	I-IV	healthy patients	0.81 [0.70-0.92]	-	-
miR-21	chr17: 59841266-59841337 [+] [82] [83]	Stroese et al. [9]	2018	exogenous cel-miR-39	serum	upregulated	I-IV	healthy patients	0.71 [0.56-0.86]	-	-
miR-99a	chr21: 16539089-16539169 [+] [172][173]	Stroese et al.[9]	2018	exogenous cel-miR-39	serum	upregulated	I-IV	healthy patients	0.72 [0.59-0.86]	-	-
miR-99b	chr19:	Stroese et	2018	exogenous cel-	serum	upregulated	I-IV	healthy patients	0.76 [0.65-0.88]	-	-

	51692612-51692681 [+] [174][175]	al.[9]		miR-39							
miR-205	chr1: 209432133-209432242 [+] [77] [78]	Traeger et al. [79]	2018	exogenous cel-miR-39	serum	upregulated	II-IV	healthy patients, CP, IPMN	0.669 [0.548–0.789]	64.3%	68.4%
miR-181c	chr19: 13874699-13874808 [+] [47] [48]	Vieira et al. [49]	2021	endogenous RNU-24 and RNU-48	plasma	upregulated	I-IV	healthy patients	0.72	-	-
miR-210	chr11: 568089-568198 [-] [87][88]	Vieira et al. [49]	2021	endogenous RNU-24 and RNU-48	plasma	upregulated	I-IV	healthy patients	0.88	-	-
miR-21-5p	chr17: 59841266-59841337 [+] [82]	Vila-Navarro et al.[93]	2019	exogenous cel-miR-39	plasma	upregulated	I-IV	healthy patients	0.862 [0.834-0.890]	62.4%	73.5%
miR-320a	chr8: 22244966-22245037 [-] [116]	Vila-Navarro et al.[93]	2019	exogenous cel-miR-39	plasma	upregulated	I-IV	healthy patients	0.849 [0.819-0.879]	83.0%	78.4%
miR-33a-3p	chr22: 41900944-41901012 [+] [118]	Vila-Navarro et al.[93]	2019	exogenous cel-miR-39	plasma	upregulated	I-IV	healthy patients	0.857 [0.828-0.886]	87.2%	76.0%
miR-93-5p	chr7: 100093768-100093847 [-] [169]	Vila-Navarro et al.[93]	2019	exogenous cel-miR-39	plasma	upregulated	I-IV	healthy patients	0.791 [0.755-0.826]	77.9%	67.3%
miR-133a	chr18: 21825698-21825785 [30][31]	Wang et al. [32]	2019	endogenous RNU6B	serum	downregulated	I-III	healthy patients	0.893	87.2%	90.6%

miR-155	chr21: 25573980-25574044 [+] [34][35]	Wang et al.[37]	2009	endogenous miRNA-16	plasma	upregulated	I-IV	healthy patients	0.67 [0.51–0.82]	-	-
miR-196a	chr17: 48632490-48632559 [-] [64]	Wang et al.[37]	2009	endogenous miRNA-16	plasma	upregulated	I-IV	healthy patients	0.69 [0.53–0.84]	-	-
miR-21	chr17: 59841266-59841337 [+] [82] [83]	Wang et al.[37]	2009	endogenous miRNA-16	plasma	upregulated	I-IV	healthy patients	0.62 [0.45–0.77	-	-
miR-210	chr11: 568089-568198 [-] [87][88]	Wang et al.[37]	2009	endogenous miRNA-16	plasma	upregulated	I-IV	healthy patients	0.65 [0.49–0.80]	-	-
miR-1246	chr 2: 176600980-176601052 [19]	Wei et al.[20]	2020	* three independent experiments	serum	upregulated	I-IV	healthy patients, pancreatic disease	0.81 [0.75 - 0.87]	-	-
miR-1290	chr1: 18897071-18897148 [-] [26]	Wei et al.[20]	2020	* three independent experiments	serum	upregulated	I-IV	healthy patients, pancreatic disease	0.91 [0.87 - 0.95]	-	-
miR-1290	chr1: 18897071-18897148 [-] [26]	Xu [27]	2021	endogenous miR-16-5p, exogenous cel-miR-39	serum	upregulated	I-IV	healthy patients	0.8857	60.9%	90.0%
miR-126-3p	chr 9: 136670602-136670686 [+] [21]	Xu et al.[22]	2016	endogenous RNU6B, miR-16	plasma	upregulated	I-IV	healthy patients	0.618 [0.550–0.682]	61.5%	60%
miR-486-5p	chr8: 41660441-41660508 [-]	Xu et al.[22]	2016	endogenous RNU6B, miR-16	plasma	upregulated	I-IV	healthy patients	0.861 [0.808–0.904]	75%	87.7%

	[138]										
miR-938	chr10: 29602264-29602346 [-] [170]	Xu et al.[22]	2016	endogenous RNU6B, miR-16	plasma	upregulated	I-IV	healthy patients	0.693 [0.628–0.753	61.5%	73%
miR-155	chr21: 25573980-25574044 [+] [34][35]	Yu et al [38]	2017	exogenous cel-miR-39	plasma	upregulated	-	healthy patients	0.822 [0.707–0.937]	-	-
miR-196a	chr17: 48632490-48632559 [-] [64]	Yu et al [38]	2017	exogenous cel-miR-39	plasma	upregulated	-	healthy patients	0.791 [0.665–0.916]	-	-
miR-20a	chr13: 91351065-91351135 [+] [80][81]	Yu et al [38]	2017	exogenous cel-miR-39	plasma	upregulated	-	healthy patients	0.884 [0.790–0.978]	-	-
miR-21	chr17: 59841266-59841337 [+] [82] [83]	Yu et al [38]	2017	exogenous cel-miR-39	plasma	upregulated	-	healthy patients	0.845 for [0.740–0.949]	-	-
miR-210	chr11: 568089-568198 [-] [87][88]	Yu et al [38]	2017	exogenous cel-miR-39	plasma	upregulated	-	healthy patients	0.687 [0.543–0.831]	-	-
miR-25	chr7: 100093560-100093643 [-] [107] [108]	Yu et al [38]	2017	exogenous cel-miR-39	plasma	upregulated	-	healthy patients	0.763 [0.635–0.891]	-	-
miR-25	chr7: 100093560-100093643 [-] [107] [108]	Yu et al.[110]	2020	endogenous let-7d, let-7g, let-7i	serum	upregulated	I-IV	healthy patients	0.939 [0.903-0.975]	82.5%	93.64%
miR-21	chr17:	Yuan et	2016	exogenous cel-	plasma	upregulated	I-IV	healthy patients	0.809(0.743-	-	-

	59841266-59841337 [+] [82] [83]	al.[86]		miR-39					0.875)		
miR-25	chr7: 100093560-100093643 [-] [107] [108]	Yuan et al.[86]	2016	exogenous cel-miR-39	plasma	upregulated	I-IV	healthy patients	0.655(0.570-0.740)	-	-
let-7b-5p	chr 22: 46113686-46113768 [2]	Zou et al. [3]	2019	exogenous cel-miR-39	serum	upregulated	I-IV	healthy patients	0.703 [0.636-0.771]	79.8%	59.8%
miR-192-5p	chr11: 64891137-64891246 [-] [60]	Zou et al. [3]	2019	exogenous cel-miR-39	serum	upregulated	I-IV	healthy patients	0.684 [0.615-0.754],	77.5%	57.0%
miR-19a-3p	chr13: 91350891-91350972 [+] [70]	Zou et al. [3]	2019	exogenous cel-miR-39	serum	upregulated	I-IV	healthy patients	0.771 [0.712-0.831]	71.3%	78.5%
miR-19b-3p	chr13: 91351192-91351278 [+] [71]	Zou et al. [3]	2019	exogenous cel-miR-39	serum	upregulated	I-IV	healthy patients	0.788 [0.729-0.846]	65.1%	81.3%
miR-223-3p	chrX: 66018870-66018979 [+] [99]	Zou et al. [3]	2019	exogenous cel-miR-39	serum	upregulated	I-IV	healthy patients	0.901 [0.861-0.941]	78.3%	91.6%
miR-25-3p	chr7: 100093560-100093643 [-][108]	Zou et al. [3]	2019	exogenous cel-miR-39	serum	upregulated	I-IV	healthy patients	0.726 [0.659-0.792]	66.7%	80.4%

Supplementary Table S3. Diagnostic performance of circulating miRNA panels. CS- clinical stage, PC –pancreatic cancer, AUC – area under the curve, CA19-9 - Carbohydrate antigen 19-9

**Markers included in the panel and their relative levels ↓/↑**	**Author**	**Year**	**Normalization control**	**Source of sample**	**CS of PC patients**	**AUC**	**Sensitivity**	**Specificity**
miR-483-3p ↑ miR-21↑	Abue et al.[84]	2015	exogenous cel-miR-39, endogenous miR-16	plasma	I-IV	0.839	-	-
miR-21↑ miR-34a↑	Alemar et al. [85]	2016	exogenous cel-miR-39	serum	I-IV	0.894 [0.771–1.0]	-	-
miR-196b↑ TIMP1↑ LCN2↑	Bartsch et al. [68]	2018	endogenous miR-24	serum	I-III	0.93 [0.8162 to 1.044]	80%	80%
miRNA-10b↑ miRNA -106b↑	Cote et al. [7]	2014	endogenous miRNA-425-5p	plasma	I-IV	-	100%	100%
miR-8073↑ miR-642↑	Fathi et al. [151]	2022	endogenous RNU6B	plasma	-	0.79	77%	78%
miR-574–3p ↑ miR-885–5p ↑ miR-144–3p↓ miR-130b-3p ↑ miR-34a-5p↑ miR-24–3p↑ miR-106b-5p↓ miR-22–5p↑ miR-451a↓ let-7d-3p↑ miR-101–3p↓ miR-26a-5p↓ miR-197–3p↑ miR-423–3p↑ miR-122–5p↑	Franklin et al [5]	2018	endogenous mean expression value normalization	plasma	I-II	0.96 [0.92-1.00]	-	-
miR-642b-3p↑ miR -885-5p↑	Ganepola et al. [102]	2014	endogenous miR-3196	plasma	IIA-IIB	0.97 [0.90-1.00]	-	-

miR- 22-3p↑								
miR-607↓<br>CA19.9↑<br>CEA↑	Jiang et al. [144]	2021	endogenous RNU6B	serum	I-IV	0.863	76.6%	83.1%
miR-16 ↑<br>miR-24↓<br>miR-27.a↓<br>miR-30a-5p↓<br>miR-323-3p↓<br>miR-20a↑<br>miR-25↓<br>miR-29c↓<br>miR-483-5p ↑	Johansen et al.[176]	2016	endogenous controls	serum	I-IV	0.7207	-	-
miR-16↑<br>miR-18.a↑<br>miR-24↓<br>miR-27a↓<br>miR-30a-5p↓<br>miR-323-3p↓<br>miR-20a↑<br>miR-25↓<br>miR-29c↓<br>miR-191↓<br>miR-345↑<br>miR-483-5p ↑	Johansen et al.[176]	2016	endogenous controls	serum	I-IV	0.7237		
miR-221↑<br>miR-375↓	Kawaguchi et al.[96]	2013		plasma	I-IV	0.762	-	-
miR-215-5p↑<br>miR-122-5p↑<br>miR-192-5p↑<br>miR-30b-5p↓<br>miR-320b↑	Khan et al.[18]	2021	exogenous UniSp6	serum	I-IV	0.811 [0.744–0.829]	-	-
miR-492↓<br>miR-663a↓	Lin et al.[140]	2014	exogenous cel-miR-39	serum	I-IV	0.869 [0.791-0.947]	85.7%	80.0%
miR-181b↑<br>miR-210↑	Liu et al. [46]	2020	endogenous RNU6B	plasma	I-IV	0.830 [0.743-0.917]	80.0%	70.0%

miR-196a↑ miR-210↑	Liu et al. [46]	2020	endogenous RNU6B	plasma	I-IV	0.888 [0.805-0.970]	87.5%	80.0%
miR-181b↑ miR-196a↑	Liu et al. [46]	2020	endogenous RNU6B	plasma	I-IV	0.944 [0.887-1.001]	92.5%	90.0%
miR-181b↑ miR-196a↑ miR-210↑	Liu et al. [46]	2020	endogenous RNU6B	plasma	I-IV	0.968 [0.924-1.011]	95.0%	97.5%
miR-16↑ miR-196a↑	Liu et al. [36]	2012	exogenous cel-miR-39	plasma	I-IV	0.895 [0.850–0.939]	-	-
miR-16↑ miR-196a↑ CA19-9↑	Liu et al. [36]	2012	exogenous cel-miR-39	plasma	I-IV	0.979 [0.962–0.996]	92.0%	95.6%,
miR-20a↑ miR-21↑ miR-24↑ miR-25↑ miR-99a↑ miR-185↑ miR-191↑	Liu et al.[177]	2012	directly normalized to the serum volume	serum	I-IV	0.985	94%	93%
miR-145↓ miR-150↓ miR-223↑ miR-636↓	Schultz et al. [178]	2014	endogenous RNU44 and RNU48	whole blood	I-IV	0.88 [0.85-0.92]	85%	73%
miR-26b↑ miR-34a↑ miR-122↑ miR-126↓ miR-145↑ miR-150↓ miR-223↑ miR-505↑ miR-636↓ miR-885-5p↑	Schultz et al. [178]	2014	endogenous RNU44 and RNU48	whole blood	I-IV	0.93 [0.89-0.96]	85%	86%
miR-196↑	Škrha et	2016	endogenous miR-	serum	-	-	94%	82%

miR-200 ↑<br>CA 19-9↑	al.[63]		191 and miR-454v					
miR-196a↑<br>miR-196b↑	Slater et al.[65]	2014	endogenous miR-24	serum	I,II,IV	1.00	100%	100%
miR-210↑<br>miR-21↑<br>CA19-9↑	Vieira et al.[49]	2021	endogenous RNU-24 and RNU-48	plasma	I-IV	1.00	100%	100%
miR-33a-3p↑<br>miR-320a↑	Vila-Navarro et al.[93]	2019	exogenous cel-miR-39	plasma	I-IV	0.906 [0.93-0.88]	89%	85%
miR-33a-3p↑<br>miR-320a↑<br>CA19-9↑	Vila-Navarro et al.[93]	2019	exogenous cel-miR-39	plasma	I-IV	0.956 [0.97-0.94]	86%	91%
miR-21↑<br>miR-210↑<br>miR-155↑<br>miR-196a↑	Wang et al.[37]	2009	endogenous miRNA-16	plasma	I-IV	0.82 [0.70–0.94]	64%	89%
miR-1290↑<br>miR-1246↑<br>CA19-9↑	Wei et al.[20]	2020	* three independent experiments	serum	I-IV	0.97 [0.96-1.00]	92.5%	93.7%
miR-25↑<br>MIC-1↑<br>CA19-9↑	Yuan et al.[86]	2016	exogenous cel-miR-39	plasma	I-IV	0.967 [0.938-0.995]	84.1%	100%
miR-21↑<br>MIC-1↑<br>CA19-9↑	Yuan et al.[86]	2016	exogenous cel-miR-39	plasma	I-IV	0.972 [0.946-0.999]	87.8%	98.9%
miR-122-5p↑<br>miR-125b-5p↑<br>miR-192-5p↑<br>miR-193b-3p↑<br>miR-221-3p↑<br>miR-27b-3p↑	Zhou et al.[179]	2018	endogenous miR-103a	plasma	I-IV	0.937 [0.894–0.979]	88.7%	89.1%
let-7b-5p↑<br>miR-192-5p↑<br>miR-19a-3p↑<br>miR-19b-3p↑<br>miR-223-3p↑	Zou et al. [3]	2019	exogenous cel-miR-39	serum	I-IV	0.978 [0.966-0.998]	93.3%	96.0%

miR-25-3p↑								

Supplementary Table S4. Diagnostic performance of circulating miRNA compared to CA19-9. CS – clinical stage, PC – pancreatic cancer, AUC –area under the curve, CA19-9 - Carbohydrate antigen 19-9

* analysis with Abcam Fireplex™ platform

miRNA	Author	Year	Normalization control	Source of sample	Expression level compared to non-cancer controls.	CS of PC patients	Compared with CA19-9 alone	AUC in panel with CA19.9	Sensitivity in panel with CA19-9	Specificity in panel with CA19-9
miR-21	Abue et al.[84]	2015	exogenous cel-miR-39,	plasma	upregulated	I-IV	worse results	-	-	-

			endogenous miR-16							
miR-483-3p	Abue et al.[84]	2015	exogenous cel-miR-39, endogenous miR-16	plasma	upregulated	I-IV	worse results	-	-	-
miR-1202	Aita et al. [16]	2021	exogenous UniSp6, exogenous spike-in mix (UniSp2, UniSp4, UniSp5)	serum	downregulated	I-III	better results	-	-	-
miR-1275	Aita et al. [16]	2021	exogenous UniSp6, exogenous spike-in mix (UniSp2, UniSp4, UniSp5)	serum	downregulated	I-III	better results	-	-	-
miR-1915-3p	Aita et al. [16]	2021	exogenous UniSp6, exogenous spike-in mix (UniSp2, UniSp4, UniSp5)	serum	upregulated	I-III	better results	-	-	-
miR-2392	Aita et al. [16]	2021	exogenous UniSp6, exogenous spike-in mix (UniSp2, UniSp4, UniSp5)	serum	downregulated	I-III	better results	-	-	-
miR-3135-b	Aita et al. [16]	2021	exogenous UniSp6, exogenous spike-in mix (UniSp2, UniSp4, UniSp5)	serum	upregulated	I-III	better results	-	-	-
miR-3679-5p	Ait. et al. [16]	2021	exogenous UniSp6, exogenous spike-in mix (UniSp2, UniSp4, UniSp5)	serum	downregulated	I-III	better results	-	-	-

miR-371b-5p	Aita et al. [16]	2021	exogenous UniSp6, exogenous spike-in mix (UniSp2, UniSp4, UniSp5)	serum	upregulated	I-III	better results	-	-	-
miR-4327	Aita et al. [16]	2021	exogenous UniSp6, exogenous spike-in mix (UniSp2, UniSp4, UniSp5)	serum	upregulated	I-III	better results	-	-	-
miR-4466	Aita et al. [16]	2021	exogenous UniSp6, exogenous spike-in mix (UniSp2, UniSp4, UniSp5)	serum	upregulated	I-III	better results	-	-	-
miR-4516	Aita et al. [16]	2021	exogenous UniSp6, exogenous spike-in mix (UniSp2, UniSp4, UniSp5)	serum	upregulated	I-III	better results	-	-	-
miR-4655-3p	Aita et al. [16]	2021	exogenous UniSp6, exogenous spike-in mix (UniSp2, UniSp4, UniSp5)	serum	upregulated	I-III	better results	-	-	-
miR-4669	Aita et al. [16]	2021	exogenous UniSp6, exogenous spike-in mix (UniSp2, UniSp4, UniSp5)	serum	downregulated	I-III	better results	-	-	-
miR-4687-3p	Aita et al. [16]	2021	exogenous UniSp6, exogenous spike-in mix (UniSp2, UniSp4, UniSp5)	serum	upregulated	I-III	better results	-	-	-
miR-5100	Aita et al.	2021	exogenous	serum	upregulated	I-III	better results	-	-	-

	[16]		UniSp6, exogenous spike-in mix (UniSp2, UniSp4, UniSp5)							
miR-574-3p	Aita et al. [16]	2021	exogenous UniSp6, exogenous spike-in mix (UniSp2, UniSp4, UniSp5)	serum	downregulated	I-III	better results	-	-	-
miR-6089	Aita et al. [16]	2021	exogenous UniSp6, exogenous spike-in mix (UniSp2, UniSp4, UniSp5)	serum	upregulated	I-III	better results	-	-	-
miR-6125	Aita et al. [16]	2021	exogenous UniSp6, exogenous spike-in mix (UniSp2, UniSp4, UniSp5)	serum	upregulated	I-III	better results	-	-	-
miR-6126	Aita et al. [16]	2021	exogenous UniSp6, exogenous spike-in mix (UniSp2, UniSp4, UniSp5)	serum	upregulated	I-III	better results	-	-	-
miR-6749-5p	Aita et al. [16]	2021	exogenous UniSp6, exogenous spike-in mix (UniSp2, UniSp4, UniSp5)	serum	downregulated	I-III	better results	-	-	-
miR-6800-5p	Aita et al. [16]	2021	exogenous UniSp6, exogenous spike-in mix (UniSp2, UniSp4, UniSp5)	serum	upregulated	I-III	better results	-	-	-
miR-6821-5p	Aita et al. [16]	2021	exogenous UniSp6,	serum	upregulated	I-III	better results	-	-	-

			exogenous spike-in mix (UniSp2, UniSp4, UniSp5)							
miR-6850-5p	Aita et al. [16]	2021	exogenous UniSp6, exogenous spike-in mix (UniSp2, UniSp4, UniSp5)	serum	upregulated	I-III	better results	-	-	-
miR-6869-5p	Aita et al. [16]	2021	exogenous UniSp6, exogenous spike-in mix (UniSp2, UniSp4, UniSp5)	serum	upregulated	I-III	better results	-	-	-
miR-7107-5p	Aita et al. [16]	2021	exogenous UniSp6, exogenous spike-in mix (UniSp2, UniSp4, UniSp5)	serum	downregulated	I-III	better results	-	-	-
miR-7110-5p	Aita et al. [16]	2021	exogenous UniSp6, exogenous spike-in mix (UniSp2, UniSp4, UniSp5)	serum	downregulated	I-III	better results	-	-	-
miR-8485	Aita et al. [16]	2021	exogenous UniSp6, exogenous spike-in mix (UniSp2, UniSp4, UniSp5)	serum	downregulated	I-III	better results	-	-	-
miR-939-5p	Aita et al. [16]	2021	exogenous UniSp6, exogenous spike-in mix (UniSp2, UniSp4, UniSp5)	serum	upregulated	I-III	better results	-	-	-
miR-182	Chen et al [55]	2014	endogenous RNU6B	plasma	upregulated	I-IV	better results	0.837 [0.769–0.894]	84.68 %	86.77 %

miR-25	Deng et al.[109]	2016	directly normalized to the serum volume	serum	upregulated	I-IV	better results	-	-	-
miR-130a-3p*	Dittmar et al. [29]	2021	endogenous miR-17–5p, miR-20a-5p, miR-93–5p	plasma	upregulated	II	worse results	0.94 [0.89-0.98]	-	-
miR-222–3p*	Dittmar et al. [29]	2021	endogenous miR-17–5p, miR-20a-5p, miR-93–5p	plasma	upregulated	II	worse results	0.92 [0.87-0.97]	-	-
miR-34a-5p*	Dittmar et al. [29]	2021	endogenous miR-17–5p, miR-20a-5p, miR-93–5p	plasma	upregulated	II	worse results	0.92 [0.86 - 0.97]	-	-
miR-22-3p	Hussein et al.[103]	2017	endogenous miR-3196	serum	upregulated	IB,IIB,IV	better results	-	-	-
miR-642b-3p	Hussein et al.[103]	2017	endogenous miR-3196	serum	upregulated	IB,IIB,IV	better results	-	-	-
miR-885-5p	Hussein et al.[103]	2017	endogenous miR-3196	serum	upregulated	IB,IIB,IV	better results	-	-	-
miR-607	Jiang et al. [144]	2021	endogenous RNU6B	serum	downregulated	I-IV	better results	-	-	-
miR-181b	Liu et al. [46]	2020	endogenous RNU6B	plasma	upregulated	I-IV	worse results	-	-	-
miR-196a	Liu et al. [46]	2020	endogenous RNU6B	plasma	upregulated	I-IV	worse results	-	-	-
miR-210	Liu et al. [46]	2020	endogenous RNU6B	plasma	upregulated	I-IV	worse results	-	-	-
miR-629	Shi et al.[150]	2018	-	serum	upregulated	I-IV	better results	0.812 [0.733 - 0.8927]	-	-
miR-205	Traeger et al. [79]	2018	exogenous cel-miR-39	serum	upregulated	II-IV	worse results	0.890 [0.782–0.995]	86.7%	93.3%
miR-210	Vieira et al. [49]	2021	endogenous RNU-24 and RNU-48	plasma	upregulated	I-IV	worse results	0.99	-	-
miR-21-5p	Vila-Navarro et al.[93]	2019	exogenous cel-miR-39	plasma	upregulated	I-IV	better results	-	-	-

miR-320a	Vila-Navarro et al.[93]	2019	exogenous cel-miR-39	plasma	upregulated	I-IV	better results	-	-	-
miR-33a-3p	Vila-Navarro et al.[93]	2019	exogenous cel-miR-39	plasma	upregulated	I-IV	better results	-	-	-
miR-93-5p	Vila-Navarro et al.[93]	2019	exogenous cel-miR-39	plasma	upregulated	I-IV	worse results	-	-	-
miR-1246	Wei et al.[20]	2020	* three independent experiments	serum	upregulated	I-IV	worse results	0.93 [0.89-0.96]	85.8 %	86.2 %
miR-1290	Wei et al.[20]	2020	* three independent experiments	serum	upregulated	I-IV	better results	0.96 [0.94-0.99]	84.2 %	96.2 %
miR-25	Yu et al.[110]	2020	endogenous let-7d, let-7g, let-7i	serum	upregulated	I-IV	better results	0.985 [0.972-0.998]	-	-
miR-21	Yuan et al.[86]	2016	exogenous cel-miR-39	plasma	upregulated	I-IV	worse results	-	-	-
miR-25	Yuan et al.[86]	2016	exogenous cel-miR-39	plasma	upregulated	I-IV	worse results	-	-	-
miR-122-5p	Zhou et al.[179]	2018	endogenous miR-103a	plasma	upregulated	I-IV	-	0.81 [0.762–0.859]	-	-
miR-125b-5p	Zhou et al.[179]	2018	endogenous miR-103a	plasma	upregulated	I-IV	-	0.646 [0.585–0.707]	-	-
miR-192-5p	Zhou et al.[179]	2018	endogenous miR-103a	plasma	upregulated	I-IV	-	0.693 [0.634–0.753]	-	-
miR-193b-3p	Zhou et al.[179]	2018	endogenous miR-103a	plasma	upregulated	I-IV	-	0.775 [0.722–0.828]	-	-
miR-221-3p	Zhou et al.[179]	2018	endogenous miR-103a	plasma	upregulated	I-IV	-	0.625 [0.562–0.688]	-	-
miR-27b-3p	Zhou et al.	2018	endogenous	plasma	upregulated	I-IV	-	0.656	-	-

	[179]		miR-103a					[0.594–0.718]		

Supplementary Table S5. Prognostic performance of circulating miRNA. OS-overall survival
*-in unresectable patients
†-in patients undergoing pancreatoduodenctomy

**miRNA**	**Author**	**Year**	**Normalization control**	**Source of sample**	**Expression level compared to non-cancer controls.**	**Prognosis with increased level of miRNA expression**	**OS with high levels of expression in months**	**OS with low levels of expression in months**	**Univariate analysis p value**	**Multivariate analysis p value**
miR-21*	Abue et al.[84]	2015	exogenous cel-miR-39, endogenous miR-16	plasma	upregulated	poor	3.0	13.8	**p=0.005**	-
let-7a	Ali et al.[180]	2015	-	plasma	downregulated	good	-	-	p=0.063	-
miR-508	Ali et al.[180]	2015	-	plasma	upregulated	poor	-	-	p=0.212	-
let-7d	Ali et al.[181]	2010	endogenous miR-16	plasma	downregulated	good	-	-	-	**p=0.0044**
miR-21	Ali et al.[181]	2010	endogenous miR-16	plasma	upregulated	poor	-	-	-	**p=0.0032**
miR-182	Chen et al [55]	2014	endogenous RNU6B	plasma	upregulated	poor	25.023, [22.122–27.878]	37.025, [32.753–41.247]	**p=0.001**	**p<0.001**
miR-200c-3p†	Gablo et al. [182]	2020	endogenous miR-93-5p	plasma	-	poor	-	-	**p =0.012**	-
miR-365a-3p†	Gablo et al. [182]	2020	endogenous miR-93-5p	plasma	-	good	-	-	**p =0.013**	-
miR-99a-5p†	Gablo et	2020	endogenous miR-	plasma	-	good	-	-	**p =0.006**	-

	al. [182]		93-5p							
miR-373	Hua et al.[125]	2017	endogenous RNU6B	serum	downregulated	good	-	-	-	**p=0.014**
miR-107	Imamura et al.[12]	2017	exogenous cel-miR-39	plasma	downregulated	good	-	-	**p=0.0038**	**p=0.0424**
miR-607†	Jiang et al. [144]	2021	endogenous RNU6B	serum	downregulated	good	-	-	**p=0.011**	**p=0.036**
miR-21-5p†	Karasek et al. [24]	2018	endogenous miR-106a	plasma	upregulated	poor	-	-	**p=0.0499**	**p=0.0379**
miR-196a	Kong et al. [183]	2011	exogenous cel-miR-39	serum	upregulalted	poor	6.1 [4.49–7.72]	12.0 [5.92–18.08]	**p = 0.007**	
miR-21	Liu et al.[177]	2012	directly normalized to the serum volume	serum	upregulated	poor	-	-	**p=0.02**	**p=0.00**
miR-744	Miyamae et al.[165]	2015	exogenous cel-miR-39	plasma	upregulated	poor	-	-	**p=0.0063**	**p=0.0007†**
miR-629	Shi et al.[150]	2018	-	serum	upregulated	poor	-	-	**p=0.001**	**p=0.006**
miR-100	Stroese et al. [9]	2018	exogenous cel-miR-39	serum	upregulated	poor	-	-	**p = 0.002**	**p = 0.046**
miR-21	Stroese et al.[9]	2018	exogenous cel-miR-39	serum	upregulated	poor	-	-	**p = 0.001**	**p = 0.004**
miR-205	Traeger et al. [79]	2018	exogenous cel-miR-39	serum	upregulated	poor	6.93	11.93	p = 0.176	-
miR-17-3p	Van der Sijde et al.[184]	2021	endogenous miR-26a-5p, miR-30b-5p	serum	-	-	-	-	**p = 0.032**	p = 0.192
miR-194-5p	Van der Sijde et al.[184]	2021	endogenous miR-26a-5p, miR-30b-5p	serum	-	-	-	-	p =0.145	-
miR-373-3p	Van der Sijde et al.[184]	2021	endogenous miR-26a-5p, miR-30b-5p	serum	-	-	-	-	p =0.141	-
miR-21	Wang et al. [185]	2013	exogenous cel-miR-39	serum	upregulated	poor	6.2	11.6	**p = 0.002**	**p = 0.008**
miR-196a	Yu et al	2017	exogenous cel-	plasma	upregulated	poor	6.3 [2.3–10.3]	12.5 [10.0–	**p=0.001**	**p=0.013**

	[38]		miR-39					15.0]		
miR-210	Yu et al [38]	2017	exogenous cel-miR-39	plasma	upregulated	good	11.7 [6.8–16.5]	6.6 [5.3–7.9]	**p = 0.003**	**p=0.021**
miR-21	Yuan et al.[86]	2016	exogenous cel-miR-39	plasma	upregulated	good	8.30 [5.69-10.91]	7.30 [5.94-8.66]	p=0.768	-
miR-25	Yuan et al.[86]	2016	exogenous cel-miR-39	plasma	upregulated	good	8.30 [4.73-11.87]	7.30[6.04-8.56]	p= 0.759	-
miR-125b-5p	Zhou et al. [179]	2018	endogenous miR-103a	plasma	upregulated	good	-	-	**p=0.004**	**p=0.011**
miR-19a-3p	Zou et al. [3]	2019	exogenous cel-miR-39	serum	upregulated	poor	-	-	**p=0.036**	**p=0.042**

References:

[1] E. S. Lee and J. M. Lee, "Imaging diagnosis of pancreatic cancer: A state-of-the-art review," *World J. Gastroenterol.*, vol. 20, no. 24, p. 7864, Jun. 2014.

[2] "miRDB Search Result." [Online]. Available: https://mirdb.org/cgi-bin/search.cgi?searchType=miRNA&full=mirbase&searchBox=MIMAT0000063. [Accessed: 26-Feb-

2023].
[3] X. Zou *et al.*, "Identification of a six-miRNA panel in serum benefiting pancreatic cancer diagnosis," *Cancer Med.*, vol. 8, no. 6, pp. 2810–2822, Jun. 2019.
[4] "miRDB Search Result." [Online]. Available: https://mirdb.org/cgi-bin/search.cgi?searchType=miRNA&full=mirbase&searchBox=MIMAT0000065. [Accessed: 26-Feb-2023].
[5] O. Franklin *et al.*, "Plasma Micro-RNA Alterations Appear Late in Pancreatic Cancer," *Ann. Surg.*, vol. 267, no. 4, p. 775, Apr. 2018.
[6] "miRDB Search Result." [Online]. Available: https://mirdb.org/cgi-bin/search.cgi?searchType=miRNA&full=mirbase&searchBox=MIMAT0000680. [Accessed: 26-Feb-2023].
[7] G. A. Cote *et al.*, "A pilot study to develop a diagnostic test for pancreatic ductal adenocarcinoma based on differential expression of select miRNA in plasma and bile," *Am. J. Gastroenterol.*, vol. 109, no. 12, pp. 1942–1952, Dec. 2014.
[8] "miRDB Search Result." [Online]. Available: https://mirdb.org/cgi-bin/search.cgi?searchType=miRNA&full=mirbase&searchBox=MIMAT0000098. [Accessed: 26-Feb-2023].
[9] A. J. Stroese, H. Ullerich, G. Koehler, V. Raetzel, N. Senninger, and S. A. Dhayat, "Circulating microRNA-99 family as liquid biopsy marker in pancreatic adenocarcinoma," *J. Cancer Res. Clin. Oncol.*, vol. 144, no. 12, pp. 2377–2390, Dec. 2018.
[10] "miRDB Search Result." [Online]. Available: https://mirdb.org/cgi-bin/search.cgi?searchType=miRNA&full=mirbase&searchBox=MIMAT0000099. [Accessed: 26-Feb-2023].
[11] "miRDB Search Result." [Online]. Available: https://mirdb.org/cgi-bin/search.cgi?searchType=miRNA&full=mirbase&searchBox=MIMAT0000104. [Accessed: 26-Feb-2023].
[12] T. Imamura *et al.*, "Depleted tumor suppressor miR-107 in plasma relates to tumor progression and is a novel therapeutic target in pancreatic cancer," *Sci. Rep.*, vol. 7, no. 1, Dec. 2017.
[13] "miRDB Search Result." [Online]. Available: https://mirdb.org/cgi-bin/search.cgi?searchType=miRNA&full=mirbase&searchBox=MIMAT0000254. [Accessed: 26-Feb-2023].
[14] "miRDB Search Result." [Online]. Available: https://mirdb.org/cgi-bin/search.cgi?searchType=miRNA&full=mirbase&searchBox=MIMAT0004556. [Accessed: 26-Feb-2023].
[15] "miRDB Search Result." [Online]. Available: https://mirdb.org/cgi-bin/search.cgi?searchType=miRNA&full=mirbase&searchBox=MIMAT0005865. [Accessed: 26-Feb-2023].
[16] A. Aita *et al.*, "Serum miRNA Profiling for Early PDAC Diagnosis and Prognosis: A Retrospective Study," *Biomedicines*, vol. 9, no. 7, Jul. 2021.
[17] "miRDB Search Result." [Online]. Available: https://mirdb.org/cgi-bin/search.cgi?searchType=miRNA&full=mirbase&searchBox=MIMAT0000421. [Accessed: 26-Feb-2023].
[18] I. A. Khan *et al.*, "Panel of serum miRNAs as potential non-invasive biomarkers for pancreatic ductal adenocarcinoma," *Sci. Rep.*, vol. 11, no. 1, Dec. 2021.
[19] "miRDB Search Result." [Online]. Available: https://mirdb.org/cgi-bin/search.cgi?searchType=miRNA&full=mirbase&searchBox=MIMAT0005898. [Accessed: 26-Feb-2023].
[20] J. Wei, L. Yang, Y. N. Wu, and J. Xu, "Serum miR-1290 and miR-1246 as Potential Diagnostic Biomarkers of Human Pancreatic Cancer," *J. Cancer*, vol. 11, no. 6, pp. 1325–1333, 2020.
[21] "miRDB Search Result." [Online]. Available: https://mirdb.org/cgi-bin/search.cgi?searchType=miRNA&full=mirbase&searchBox=MIMAT0000445. [Accessed: 26-Feb-2023].
[22] J. Xu *et al.*, "Plasma miRNAs Effectively Distinguish Patients With Pancreatic Cancer From Controls: A Multicenter Study," *Ann. Surg.*, vol. 263, no. 6, pp. 1173–1179, 2016.

[23] "miRDB Search Result." [Online]. Available: https://mirdb.org/cgi-bin/search.cgi?searchType=miRNA&full=mirbase&searchBox=MIMAT0000444. [Accessed: 26-Feb-2023].
[24] P. Karasek *et al.*, "Pre-operative Plasma miR-21-5p Is a Sensitive Biomarker and Independent Prognostic Factor in Patients with Pancreatic Ductal Adenocarcinoma Undergoing Surgical Resection," *Cancer Genomics Proteomics*, vol. 15, no. 4, pp. 321–327, Jul. 2018.
[25] "miRDB Search Result." [Online]. Available: https://mirdb.org/cgi-bin/search.cgi?searchType=miRNA&full=mirbase&searchBox=MIMAT0005929. [Accessed: 26-Feb-2023].
[26] "miRDB Search Result." [Online]. Available: https://mirdb.org/cgi-bin/search.cgi?searchType=miRNA&full=mirbase&searchBox=MIMAT0005880. [Accessed: 26-Feb-2023].
[27] L. Xu, Y. Cai, X. Chen, Y. Zhu, and J. Cai, "Circulating MiR-1290 as a potential diagnostic and disease monitoring biomarker of human gastrointestinal tumors," *BMC Cancer*, vol. 21, no. 1, Dec. 2021.
[28] "miRDB Search Result." [Online]. Available: https://mirdb.org/cgi-bin/search.cgi?searchType=miRNA&full=mirbase&searchBox=MIMAT0000425. [Accessed: 26-Feb-2023].
[29] R. L. Dittmar *et al.*, "Plasma MicroRNA Biomarkers in Limited Volume Samples for Detection of Early Stage Pancreatic Cancer," *Cancer Prev. Res. (Phila).*, vol. 14, no. 7, p. 729, Jul. 2021.
[30] "miRDB Search Result." [Online]. Available: https://mirdb.org/cgi-bin/search.cgi?searchType=miRNA&full=mirbase&searchBox=MIMAT0026478. [Accessed: 26-Feb-2023].
[31] "miRDB Search Result." [Online]. Available: https://mirdb.org/cgi-bin/search.cgi?searchType=miRNA&full=mirbase&searchBox=MIMAT0000427. [Accessed: 26-Feb-2023].
[32] Z. Wang, "Diagnostic performance for declined microRNA-133a in pancreatic cancer," *J. Cell. Biochem.*, vol. 121, no. 8–9, pp. 3882–3886, Aug. 2019.
[33] "miRDB Search Result." [Online]. Available: https://mirdb.org/cgi-bin/search.cgi?searchType=miRNA&full=mirbase&searchBox=MIMAT0000436. [Accessed: 26-Feb-2023].
[34] "miRDB Search Result." [Online]. Available: https://mirdb.org/cgi-bin/search.cgi?searchType=miRNA&full=mirbase&searchBox=MIMAT0000646. [Accessed: 26-Feb-2023].
[35] "miRDB Search Result." [Online]. Available: https://mirdb.org/cgi-bin/search.cgi?searchType=miRNA&full=mirbase&searchBox=MIMAT0004658. [Accessed: 26-Feb-2023].
[36] J. Liu *et al.*, "Combination of plasma microRNAs with serum CA19-9 for early detection of pancreatic cancer," *Int. J. cancer*, vol. 131, no. 3, pp. 683–691, Aug. 2012.
[37] J. Wang *et al.*, "MicroRNAs in plasma of pancreatic ductal adenocarcinoma patients as novel blood-based biomarkers of disease," *Cancer Prev. Res. (Phila).*, vol. 2, no. 9, pp. 807–813, Sep. 2009.
[38] Q. Yu *et al.*, "Evaluation of Plasma MicroRNAs as Diagnostic and Prognostic Biomarkers in Pancreatic Adenocarcinoma: miR-196a and miR-210 Could Be Negative and Positive Prognostic Markers, Respectively," *Biomed Res. Int.*, vol. 2017, 2017.
[39] "miRDB Search Result." [Online]. Available: https://mirdb.org/cgi-bin/search.cgi?searchType=miRNA&full=mirbase&searchBox=MIMAT0000069. [Accessed: 26-Feb-2023].
[40] "miRDB Search Result." [Online]. Available: https://mirdb.org/cgi-bin/search.cgi?searchType=miRNA&full=mirbase&searchBox=MIMAT0004489. [Accessed: 26-Feb-2023].
[41] "miRDB Search Result." [Online]. Available: https://mirdb.org/cgi-bin/search.cgi?searchType=miRNA&full=mirbase&searchBox=MIMAT0000070. [Accessed: 26-Feb-2023].
[42] "miRDB Search Result." [Online]. Available: https://mirdb.org/cgi-bin/search.cgi?searchType=miRNA&full=mirbase&searchBox=MIMAT0000256. [Accessed: 26-Feb-

2023].
[43] "miRDB Search Result." [Online]. Available: https://mirdb.org/cgi-bin/search.cgi?searchType=miRNA&full=mirbase&searchBox=MIMAT0000270. [Accessed: 26-Feb-2023].
[44] "miRDB Search Result." [Online]. Available: https://mirdb.org/cgi-bin/search.cgi?searchType=miRNA&full=mirbase&searchBox=MIMAT0000257. [Accessed: 26-Feb-2023].
[45] "miRDB Search Result." [Online]. Available: https://mirdb.org/cgi-bin/search.cgi?searchType=miRNA&full=mirbase&searchBox=MIMAT0022692. [Accessed: 26-Feb-2023].
[46] G. Liu, C. Shao, A. Li, X. Zhang, X. Guo, and J. Li, "Diagnostic Value of Plasma miR-181b, miR-196a, and miR-210 Combination in Pancreatic Cancer," *Gastroenterol. Res. Pract.*, vol. 2020, 2020.
[47] "miRDB Search Result." [Online]. Available: https://mirdb.org/cgi-bin/search.cgi?searchType=miRNA&full=mirbase&searchBox=MIMAT0000258. [Accessed: 26-Feb-2023].
[48] "miRDB Search Result." [Online]. Available: https://mirdb.org/cgi-bin/search.cgi?searchType=miRNA&full=mirbase&searchBox=MIMAT0004559. [Accessed: 26-Feb-2023].
[49] N. F. Vieira *et al.*, "The role of circulating miRNAs and CA19-9 in pancreatic cancer diagnosis," *Oncotarget*, vol. 12, no. 17, pp. 1638–1650, Aug. 2021.
[50] "miRDB Search Result." [Online]. Available: https://mirdb.org/cgi-bin/search.cgi?searchType=miRNA&full=mirbase&searchBox=MIMAT0002821. [Accessed: 26-Feb-2023].
[51] "miRDB Search Result." [Online]. Available: https://mirdb.org/cgi-bin/search.cgi?searchType=miRNA&full=mirbase&searchBox=MIMAT0026608. [Accessed: 26-Feb-2023].
[52] M. Akamatsu *et al.*, "Specific MAPK-Associated MicroRNAs in Serum Differentiate Pancreatic Cancer from Autoimmune Pancreatitis," *PLoS One*, vol. 11, no. 7, Jul. 2016.
[53] "miRDB Search Result." [Online]. Available: https://mirdb.org/cgi-bin/search.cgi?searchType=miRNA&full=mirbase&searchBox=MIMAT0000260. [Accessed: 26-Feb-2023].
[54] "miRDB Search Result." [Online]. Available: https://mirdb.org/cgi-bin/search.cgi?searchType=miRNA&full=mirbase&searchBox=MIMAT0000259. [Accessed: 26-Feb-2023].
[55] Q. Chen, L. Yang, • Yilei, X. • Jianxin, • Z., and Z. Li, "Circulating microRNA-182 in plasma and its potential diagnostic and prognostic value for pancreatic cancer," *Med. Oncol. 2014 3111*, vol. 31, no. 11, pp. 1–8, Oct. 2014.
[56] "miRDB Search Result." [Online]. Available: https://mirdb.org/cgi-bin/search.cgi?searchType=miRNA&full=mirbase&searchBox=MIMAT0000072. [Accessed: 26-Feb-2023].
[57] "miRDB Search Result." [Online]. Available: https://mirdb.org/cgi-bin/search.cgi?searchType=miRNA&full=mirbase&searchBox=MIMAT0002891. [Accessed: 26-Feb-2023].
[58] R. Morimura *et al.*, "Novel diagnostic value of circulating miR-18a in plasma of patients with pancreatic cancer," *Br. J. Cancer*, vol. 105, no. 11, pp. 1733–1740, Nov. 2011.
[59] "miRDB Search Result." [Online]. Available: https://mirdb.org/cgi-bin/search.cgi?searchType=miRNA&full=mirbase&searchBox=MIMAT0007892. [Accessed: 26-Feb-2023].
[60] "miRDB Search Result." [Online]. Available: https://mirdb.org/cgi-bin/search.cgi?searchType=miRNA&full=mirbase&searchBox=MIMAT0000222. [Accessed: 26-Feb-2023].
[61] "miRDB Search Result." [Online]. Available: https://mirdb.org/cgi-bin/search.cgi?searchType=miRNA&full=mirbase&searchBox=MIMAT0004767. [Accessed: 26-Feb-2023].

[62] "miRDB Search Result." [Online]. Available: https://mirdb.org/cgi-bin/search.cgi?searchType=miRNA&full=mirbase&searchBox=MIMAT0002819. [Accessed: 26-Feb-2023].
[63] P. Škrha *et al.*, "Serum microRNA-196 and microRNA-200 in pancreatic ductal adenocarcinoma of patients with diabetes mellitus," *Pancreatology*, vol. 16, no. 5, pp. 839–843, Sep. 2016.
[64] "miRDB Search Result." [Online]. Available: https://mirdb.org/cgi-bin/search.cgi?searchType=miRNA&full=mirbase&searchBox=MIMAT0000226. [Accessed: 26-Feb-2023].
[65] E. P. Slater *et al.*, "MicroRNA-196a and -196b as Potential Biomarkers for the Early Detection of Familial Pancreatic Cancer," *Transl. Oncol.*, vol. 7, no. 4, pp. 464–471, 2014.
[66] "miRDB Search Result." [Online]. Available: https://mirdb.org/cgi-bin/search.cgi?searchType=miRNA&full=mirbase&searchBox=MIMAT0001080. [Accessed: 26-Feb-2023].
[67] "miRDB Search Result." [Online]. Available: https://mirdb.org/cgi-bin/search.cgi?searchType=miRNA&full=mirbase&searchBox=MIMAT0009201. [Accessed: 26-Feb-2023].
[68] D. K. Bartsch *et al.*, "The Combination of MiRNA-196b, LCN2, and TIMP1 is a Potential Set of Circulating Biomarkers for Screening Individuals at Risk for Familial Pancreatic Cancer," *J. Clin. Med.*, vol. 7, no. 10, Oct. 2018.
[69] "miRDB Search Result." [Online]. Available: https://mirdb.org/cgi-bin/search.cgi?searchType=miRNA&full=mirbase&searchBox=MIMAT0000227. [Accessed: 26-Feb-2023].
[70] "miRDB Search Result." [Online]. Available: https://mirdb.org/cgi-bin/search.cgi?searchType=miRNA&full=mirbase&searchBox=MIMAT0000073. [Accessed: 26-Feb-2023].
[71] "miRDB Search Result." [Online]. Available: https://mirdb.org/cgi-bin/search.cgi?searchType=miRNA&full=mirbase&searchBox=MIMAT0000074. [Accessed: 26-Feb-2023].
[72] "miRDB Search Result." [Online]. Available: https://mirdb.org/cgi-bin/search.cgi?searchType=miRNA&full=mirbase&searchBox=MIMAT0001620. [Accessed: 26-Feb-2023].
[73] "miRDB Search Result." [Online]. Available: https://mirdb.org/cgi-bin/search.cgi?searchType=miRNA&full=mirbase&searchBox=MIMAT0000682. [Accessed: 26-Feb-2023].
[74] A. Li *et al.*, "Pancreatic cancers epigenetically silence SIP1 and hypomethylate and overexpress miR-200a/200b in association with elevated circulating miR-200a and miR-200b levels," *Cancer Res.*, vol. 70, no. 13, pp. 5226–5237, Jul. 2010.
[75] "miRDB Search Result." [Online]. Available: https://mirdb.org/cgi-bin/search.cgi?searchType=miRNA&full=mirbase&searchBox=MIMAT0004571. [Accessed: 26-Feb-2023].
[76] "miRDB Search Result." [Online]. Available: https://mirdb.org/cgi-bin/search.cgi?searchType=miRNA&full=mirbase&searchBox=MIMAT0000318. [Accessed: 26-Feb-2023].
[77] "miRDB Search Result." [Online]. Available: https://mirdb.org/cgi-bin/search.cgi?searchType=miRNA&full=mirbase&searchBox=MIMAT0000266. [Accessed: 26-Feb-2023].
[78] "miRDB Search Result." [Online]. Available: https://mirdb.org/cgi-bin/search.cgi?searchType=miRNA&full=mirbase&searchBox=MIMAT0009197. [Accessed: 26-Feb-2023].
[79] M. Michael Traeger *et al.*, "The ambiguous role of microRNA-205 and its clinical potential in pancreatic ductal adenocarcinoma," *J. Cancer Res. Clin. Oncol.*, vol. 144, no. 12, pp. 2419–2431, Dec. 2018.
[80] "miRDB Search Result." [Online]. Available: https://mirdb.org/cgi-bin/search.cgi?searchType=miRNA&full=mirbase&searchBox=MIMAT0004493. [Accessed: 26-Feb-

2023].
[81] "miRDB Search Result." [Online]. Available: https://mirdb.org/cgi-bin/search.cgi?searchType=miRNA&full=mirbase&searchBox=MIMAT0000075. [Accessed: 26-Feb-2023].
[82] "miRDB Search Result." [Online]. Available: https://mirdb.org/cgi-bin/search.cgi?searchType=miRNA&full=mirbase&searchBox=MIMAT0000076. [Accessed: 26-Feb-2023].
[83] "miRDB Search Result." [Online]. Available: https://mirdb.org/cgi-bin/search.cgi?searchType=miRNA&full=mirbase&searchBox=MIMAT0004494. [Accessed: 26-Feb-2023].
[84] M. Abue *et al.*, "Circulating miR-483-3p and miR-21 is highly expressed in plasma of pancreatic cancer," *Int. J. Oncol.*, vol. 46, no. 2, p. 539, Feb. 2015.
[85] B. Alemar *et al.*, "miRNA-21 and miRNA-34a Are Potential Minimally Invasive Biomarkers for the Diagnosis of Pancreatic Ductal Adenocarcinoma," *Pancreas*, vol. 45, no. 1, pp. 84–92, Jan. 2016.
[86] W. Yuan *et al.*, "New combined microRNA and protein plasmatic biomarker panel for pancreatic cancer," *Oncotarget*, vol. 7, no. 48, pp. 80033–80045, 2016.
[87] "miRDB Search Result." [Online]. Available: https://mirdb.org/cgi-bin/search.cgi?searchType=miRNA&full=mirbase&searchBox=MIMAT0026475. [Accessed: 26-Feb-2023].
[88] "miRDB Search Result." [Online]. Available: https://mirdb.org/cgi-bin/search.cgi?searchType=miRNA&full=mirbase&searchBox=MIMAT0000267. [Accessed: 26-Feb-2023].
[89] "miRDB Search Result." [Online]. Available: https://mirdb.org/cgi-bin/search.cgi?searchType=miRNA&full=mirbase&searchBox=MIMAT0000269. [Accessed: 26-Feb-2023].
[90] "miRDB Search Result." [Online]. Available: https://mirdb.org/cgi-bin/search.cgi?searchType=miRNA&full=mirbase&searchBox=MIMAT0022695. [Accessed: 26-Feb-2023].
[91] "miRDB Search Result." [Online]. Available: https://mirdb.org/cgi-bin/search.cgi?searchType=miRNA&full=mirbase&searchBox=MIMAT0000272. [Accessed: 26-Feb-2023].
[92] K. Qu *et al.*, "Circulating miRNA-21-5p as a diagnostic biomarker for pancreatic cancer: evidence from comprehensive miRNA expression profiling analysis and clinical validation," *Sci. Rep.*, vol. 7, no. 1, Dec. 2017.
[93] E. Vila-Navarro *et al.*, "Novel Circulating miRNA Signatures for Early Detection of Pancreatic Neoplasia," *Clin. Transl. Gastroenterol.*, vol. 10, no. 4, Apr. 2019.
[94] "miRDB Search Result." [Online]. Available: https://mirdb.org/cgi-bin/search.cgi?searchType=miRNA&full=mirbase&searchBox=MIMAT0004568. [Accessed: 26-Feb-2023].
[95] "miRDB Search Result." [Online]. Available: https://mirdb.org/cgi-bin/search.cgi?searchType=miRNA&full=mirbase&searchBox=MIMAT0000278. [Accessed: 26-Feb-2023].
[96] T. Kawaguchi *et al.*, "Clinical impact of circulating miR-221 in plasma of patients with pancreatic cancer," *Br. J. Cancer*, vol. 108, no. 2, pp. 361–369, Feb. 2013.
[97] "miRDB Search Result." [Online]. Available: https://mirdb.org/cgi-bin/search.cgi?searchType=miRNA&full=mirbase&searchBox=MIMAT0000279. [Accessed: 26-Feb-2023].
[98] "miRDB Search Result." [Online]. Available: https://mirdb.org/cgi-bin/search.cgi?searchType=miRNA&full=mirbase&searchBox=MIMAT0004570. [Accessed: 26-Feb-2023].
[99] "miRDB Search Result." [Online]. Available: https://mirdb.org/cgi-bin/search.cgi?searchType=miRNA&full=mirbase&searchBox=MIMAT0000280. [Accessed: 26-Feb-2023].
[100] S. Komatsu *et al.*, "Malignant potential in pancreatic neoplasm; new insights provided by circulating miR-223 in plasma," *Expert Opin. Biol. Ther.*, vol. 15, no. 6, pp. 773–785, Jun. 2015.

[101] "miRDB Search Result." [Online]. Available: https://mirdb.org/cgi-bin/search.cgi?searchType=miRNA&full=mirbase&searchBox=MIMAT0000077. [Accessed: 26-Feb-2023].
[102] G. A. Ganepola, J. R. Rutledge, P. Suman, A. Yiengpruksawan, and D. H. Chang, "Novel blood-based microRNA biomarker panel for early diagnosis of pancreatic cancer," *World J. Gastrointest. Oncol.*, vol. 6, no. 1, pp. 22–33, Jan. 2014.
[103] N. A. E. M. Hussein, Z. A. E. Kholy, M. M. Anwar, M. A. Ahmad, and S. M. Ahmad, "Plasma miR-22-3p, miR-642b-3p and miR-885-5p as diagnostic biomarkers for pancreatic cancer," *J. Cancer Res. Clin. Oncol.*, vol. 143, no. 1, pp. 83–93, Jan. 2017.
[104] "miRDB Search Result." [Online]. Available: https://mirdb.org/cgi-bin/search.cgi?searchType=miRNA&full=mirbase&searchBox=MIMAT0004495. [Accessed: 26-Feb-2023].
[105] "miRDB Search Result." [Online]. Available: https://mirdb.org/cgi-bin/search.cgi?searchType=miRNA&full=mirbase&searchBox=MIMAT0019043. [Accessed: 26-Feb-2023].
[106] "miRDB Search Result." [Online]. Available: https://mirdb.org/cgi-bin/search.cgi?searchType=miRNA&full=mirbase&searchBox=MIMAT0000080. [Accessed: 26-Feb-2023].
[107] "miRDB Search Result." [Online]. Available: https://mirdb.org/cgi-bin/search.cgi?searchType=miRNA&full=mirbase&searchBox=MIMAT0004498. [Accessed: 26-Feb-2023].
[108] "miRDB Search Result." [Online]. Available: https://mirdb.org/cgi-bin/search.cgi?searchType=miRNA&full=mirbase&searchBox=MIMAT0000081. [Accessed: 26-Feb-2023].
[109] T. Deng *et al.*, "Identification of Circulating MiR-25 as a Potential Biomarker for Pancreatic Cancer Diagnosis," *Cell. Physiol. Biochem.*, vol. 39, no. 5, pp. 1716–1722, Nov. 2016.
[110] Y. Yu, Y. Tong, A. Zhong, Y. Wang, R. Lu, and L. Guo, "Identification of Serum microRNA-25 as a novel biomarker for pancreatic cancer," *Medicine (Baltimore).*, vol. 99, no. 52, Dec. 2020.
[111] "miRDB Search Result." [Online]. Available: https://mirdb.org/cgi-bin/search.cgi?searchType=miRNA&full=mirbase&searchBox=MIMAT0000082. [Accessed: 26-Feb-2023].
[112] "miRDB Search Result." [Online]. Available: https://mirdb.org/cgi-bin/search.cgi?searchType=miRNA&full=mirbase&searchBox=MIMAT0000420. [Accessed: 26-Feb-2023].
[113] "miRDB Search Result." [Online]. Available: https://mirdb.org/cgi-bin/search.cgi?searchType=miRNA&full=mirbase&searchBox=MIMAT0000244. [Accessed: 26-Feb-2023].
[114] "miRDB Search Result." [Online]. Available: https://mirdb.org/cgi-bin/search.cgi?searchType=miRNA&full=mirbase&searchBox=MIMAT0004674. [Accessed: 26-Feb-2023].
[115] "miRDB Search Result." [Online]. Available: https://mirdb.org/cgi-bin/search.cgi?searchType=miRNA&full=mirbase&searchBox=MIMAT0018985. [Accessed: 26-Feb-2023].
[116] "miRDB Search Result." [Online]. Available: https://mirdb.org/cgi-bin/search.cgi?searchType=miRNA&full=mirbase&searchBox=MIMAT0000510. [Accessed: 26-Feb-2023].
[117] "miRDB Search Result." [Online]. Available: https://mirdb.org/cgi-bin/search.cgi?searchType=miRNA&full=mirbase&searchBox=MIMAT0005792. [Accessed: 26-Feb-2023].
[118] "miRDB Search Result." [Online]. Available: https://mirdb.org/cgi-bin/search.cgi?searchType=miRNA&full=mirbase&searchBox=MIMAT0004506. [Accessed: 26-Feb-2023].
[119] "miRDB Search Result." [Online]. Available: https://mirdb.org/cgi-bin/search.cgi?searchType=miRNA&full=mirbase&searchBox=MIMAT0000255. [Accessed: 26-Feb-

2023].
[120] "miRDB Search Result." [Online]. Available: https://mirdb.org/cgi-bin/search.cgi?searchType=miRNA&full=mirbase&searchBox=MIMAT0004557. [Accessed: 26-Feb-2023].
[121] "miRDB Search Result." [Online]. Available: https://mirdb.org/cgi-bin/search.cgi?searchType=miRNA&full=mirbase&searchBox=MIMAT0018104. [Accessed: 26-Feb-2023].
[122] "miRDB Search Result." [Online]. Available: https://mirdb.org/cgi-bin/search.cgi?searchType=miRNA&full=mirbase&searchBox=MIMAT0019892. [Accessed: 26-Feb-2023].
[123] "miRDB Search Result." [Online]. Available: https://mirdb.org/cgi-bin/search.cgi?searchType=miRNA&full=mirbase&searchBox=MIMAT0000725. [Accessed: 26-Feb-2023].
[124] "miRDB Search Result." [Online]. Available: https://mirdb.org/cgi-bin/search.cgi?searchType=miRNA&full=mirbase&searchBox=MIMAT0000726. [Accessed: 26-Feb-2023].
[125] Y. Hua *et al.*, "Low serum miR-373 predicts poor prognosis in patients with pancreatic cancer," *Cancer Biomark.*, vol. 20, no. 1, pp. 95–100, 2017.
[126] "miRDB Search Result." [Online]. Available: https://mirdb.org/cgi-bin/search.cgi?searchType=miRNA&full=mirbase&searchBox=MIMAT0000728. [Accessed: 26-Feb-2023].
[127] "miRDB Search Result." [Online]. Available: https://mirdb.org/cgi-bin/search.cgi?searchType=miRNA&full=mirbase&searchBox=MIMAT0001340. [Accessed: 26-Feb-2023].
[128] "miRDB Search Result." [Online]. Available: https://mirdb.org/cgi-bin/search.cgi?searchType=miRNA&full=mirbase&searchBox=MIMAT0001536. [Accessed: 26-Feb-2023].
[129] W. T. Huang, T. S. Lin, J. Y. Wu, J. M. Hong, Y. L. Chen, and F. N. Qiu, "Evaluation of miR-429 as a novel serum biomarker for pancreatic ductal adenocarcinoma and analysis its tumor suppressor function and target genes," *Eur. Rev. Med. Pharmacol. Sci.*, vol. 26, no. 13, pp. 4638–4653, 2022.
[130] "miRDB Search Result." [Online]. Available: https://mirdb.org/cgi-bin/search.cgi?searchType=miRNA&full=mirbase&searchBox=MIMAT0016889. [Accessed: 26-Feb-2023].
[131] "miRDB Search Result." [Online]. Available: https://mirdb.org/cgi-bin/search.cgi?searchType=miRNA&full=mirbase&searchBox=MIMAT0018993. [Accessed: 26-Feb-2023].
[132] "miRDB Search Result." [Online]. Available: https://mirdb.org/cgi-bin/search.cgi?searchType=miRNA&full=mirbase&searchBox=MIMAT0019053. [Accessed: 26-Feb-2023].
[133] "miRDB Search Result." [Online]. Available: https://mirdb.org/cgi-bin/search.cgi?searchType=miRNA&full=mirbase&searchBox=MIMAT0001631. [Accessed: 26-Feb-2023].
[134] "miRDB Search Result." [Online]. Available: https://mirdb.org/cgi-bin/search.cgi?searchType=miRNA&full=mirbase&searchBox=MIMAT0019722. [Accessed: 26-Feb-2023].
[135] "miRDB Search Result." [Online]. Available: https://mirdb.org/cgi-bin/search.cgi?searchType=miRNA&full=mirbase&searchBox=MIMAT0019749. [Accessed: 26-Feb-2023].
[136] "miRDB Search Result." [Online]. Available: https://mirdb.org/cgi-bin/search.cgi?searchType=miRNA&full=mirbase&searchBox=MIMAT0019775. [Accessed: 26-Feb-2023].
[137] "miRDB Search Result." [Online]. Available: https://mirdb.org/cgi-bin/search.cgi?searchType=miRNA&full=mirbase&searchBox=MIMAT0002173. [Accessed: 26-Feb-2023].
[138] "miRDB Search Result." [Online]. Available: https://mirdb.org/cgi-bin/search.cgi?searchType=miRNA&full=mirbase&searchBox=MIMAT0002177. [Accessed: 26-Feb-

2023].
[139] "miRDB Search Result." [Online]. Available: https://mirdb.org/cgi-bin/search.cgi?searchType=miRNA&full=mirbase&searchBox=MIMAT0002812. [Accessed: 26-Feb-2023].
[140] M. S. Lin, W. C. Chen, J. X. Huang, H. J. Gao, and H. H. Sheng, "Aberrant expression of microRNAs in serum may identify individuals with pancreatic cancer," *Int. J. Clin. Exp. Med.*, vol. 7, no. 12, pp. 5226–5234, Dec. 2014.
[141] "miRDB Search Result." [Online]. Available: https://mirdb.org/cgi-bin/search.cgi?searchType=miRNA&full=mirbase&searchBox=MIMAT0022259. [Accessed: 26-Feb-2023].
[142] "miRDB Search Result." [Online]. Available: https://mirdb.org/cgi-bin/search.cgi?searchType=miRNA&full=mirbase&searchBox=MIMAT0003239. [Accessed: 26-Feb-2023].
[143] "miRDB Search Result." [Online]. Available: https://mirdb.org/cgi-bin/search.cgi?searchType=miRNA&full=mirbase&searchBox=MIMAT0003275. [Accessed: 26-Feb-2023].
[144] D. Jiang, X. Yuan, J. Ni, L. Shen, M. Cai, and L. Xu, "Low Serum miR-607 Level as a Potential Diagnostic and Prognostic Biomarker in Patients of Pancreatic Ductal Adenocarcinoma: A Preliminary Study," *Can. J. Gastroenterol. Hepatol.*, vol. 2021, 2021.
[145] "miRDB Search Result." [Online]. Available: https://mirdb.org/cgi-bin/search.cgi?searchType=miRNA&full=mirbase&searchBox=MIMAT0023714. [Accessed: 26-Feb-2023].
[146] "miRDB Search Result." [Online]. Available: https://mirdb.org/cgi-bin/search.cgi?searchType=miRNA&full=mirbase&searchBox=MIMAT0024598. [Accessed: 26-Feb-2023].
[147] "miRDB Search Result." [Online]. Available: https://mirdb.org/cgi-bin/search.cgi?searchType=miRNA&full=mirbase&searchBox=MIMAT0024599. [Accessed: 26-Feb-2023].
[148] "miRDB Search Result." [Online]. Available: https://mirdb.org/cgi-bin/search.cgi?searchType=miRNA&full=mirbase&searchBox=MIMAT0004810. [Accessed: 26-Feb-2023].
[149] "miRDB Search Result." [Online]. Available: https://mirdb.org/cgi-bin/search.cgi?searchType=miRNA&full=mirbase&searchBox=MIMAT0003298. [Accessed: 26-Feb-2023].
[150] W. Shi, Y. Lu, R. Gong, J. J. Sun, and G. Liu, "Serum miR-629 is a novel molecular marker for diagnosis and the prognosis of pancreatic cancer," *Eur. Rev. Med. Pharmacol. Sci.*, vol. 22, no. 16, pp. 5187–5193, 2018.
[151] M. Fathi, H. A. Aghdaie, S. Ghafouri-Fard, and R. Shams, "Evaluation of potential of miR-8073 and miR-642 as diagnostic markers in pancreatic cancer," *Mol. Biol. Rep.*, vol. 49, no. 7, pp. 6475–6481, Jul. 2022.
[152] "miRDB Search Result." [Online]. Available: https://mirdb.org/cgi-bin/search.cgi?searchType=miRNA&full=mirbase&searchBox=MIMAT0018444. [Accessed: 26-Feb-2023].
[153] "miRDB Search Result." [Online]. Available: https://mirdb.org/cgi-bin/search.cgi?searchType=miRNA&full=mirbase&searchBox=MIMAT0003326. [Accessed: 26-Feb-2023].
[154] "miRDB Search Result." [Online]. Available: https://mirdb.org/cgi-bin/search.cgi?searchType=miRNA&full=mirbase&searchBox=MIMAT0027398. [Accessed: 26-Feb-2023].
[155] "miRDB Search Result." [Online]. Available: https://mirdb.org/cgi-bin/search.cgi?searchType=miRNA&full=mirbase&searchBox=MIMAT0027500. [Accessed: 26-Feb-2023].
[156] "miRDB Search Result." [Online]. Available: https://mirdb.org/cgi-bin/search.cgi?searchType=miRNA&full=mirbase&searchBox=MIMAT0027542. [Accessed: 26-Feb-2023].

[157] “miRDB Search Result.” [Online]. Available: https://mirdb.org/cgi-bin/search.cgi?searchType=miRNA&full=mirbase&searchBox=MIMAT0027600. [Accessed: 26-Feb-2023].
[158] “miRDB Search Result.” [Online]. Available: https://mirdb.org/cgi-bin/search.cgi?searchType=miRNA&full=mirbase&searchBox=MIMAT0027638. [Accessed: 26-Feb-2023].
[159] “miRDB Search Result.” [Online]. Available: https://mirdb.org/cgi-bin/search.cgi?searchType=miRNA&full=mirbase&searchBox=MIMAT0000252. [Accessed: 26-Feb-2023].
[160] “miRDB Search Result.” [Online]. Available: https://mirdb.org/cgi-bin/search.cgi?searchType=miRNA&full=mirbase&searchBox=MIMAT0004553. [Accessed: 26-Feb-2023].
[161] “miRDB Search Result.” [Online]. Available: https://mirdb.org/cgi-bin/search.cgi?searchType=miRNA&full=mirbase&searchBox=MIMAT0028111. [Accessed: 26-Feb-2023].
[162] “miRDB Search Result.” [Online]. Available: https://mirdb.org/cgi-bin/search.cgi?searchType=miRNA&full=mirbase&searchBox=MIMAT0028117. [Accessed: 26-Feb-2023].
[163] “miRDB Search Result.” [Online]. Available: https://mirdb.org/cgi-bin/search.cgi?searchType=miRNA&full=mirbase&searchBox=MIMAT0004945. [Accessed: 26-Feb-2023].
[164] “miRDB Search Result.” [Online]. Available: https://mirdb.org/cgi-bin/search.cgi?searchType=miRNA&full=mirbase&searchBox=MIMAT0004946. [Accessed: 26-Feb-2023].
[165] M. Miyamae *et al.*, “Plasma microRNA profiles: identification of miR-744 as a novel diagnostic and prognostic biomarker in pancreatic cancer,” *Br. J. Cancer*, vol. 113, no. 10, pp. 1467–1476, Nov. 2015.
[166] “miRDB Search Result.” [Online]. Available: https://mirdb.org/cgi-bin/search.cgi?searchType=miRNA&full=mirbase&searchBox=MIMAT0031000. [Accessed: 26-Feb-2023].
[167] “miRDB Search Result.” [Online]. Available: https://mirdb.org/cgi-bin/search.cgi?searchType=miRNA&full=mirbase&searchBox=MIMAT0033692. [Accessed: 26-Feb-2023].
[168] “miRDB Search Result.” [Online]. Available: https://mirdb.org/cgi-bin/search.cgi?searchType=miRNA&full=mirbase&searchBox=MIMAT0004947. [Accessed: 26-Feb-2023].
[169] “miRDB Search Result.” [Online]. Available: https://mirdb.org/cgi-bin/search.cgi?searchType=miRNA&full=mirbase&searchBox=MIMAT0000093. [Accessed: 26-Feb-2023].
[170] “miRDB Search Result.” [Online]. Available: https://mirdb.org/cgi-bin/search.cgi?searchType=miRNA&full=mirbase&searchBox=MIMAT0004981. [Accessed: 26-Feb-2023].
[171] “miRDB Search Result.” [Online]. Available: https://mirdb.org/cgi-bin/search.cgi?searchType=miRNA&full=mirbase&searchBox=MIMAT0004982. [Accessed: 26-Feb-2023].
[172] “miRDB Search Result.” [Online]. Available: https://mirdb.org/cgi-bin/search.cgi?searchType=miRNA&full=mirbase&searchBox=MIMAT0000097. [Accessed: 26-Feb-2023].
[173] “miRDB Search Result.” [Online]. Available: https://mirdb.org/cgi-bin/search.cgi?searchType=miRNA&full=mirbase&searchBox=MIMAT0004511. [Accessed: 26-Feb-2023].
[174] “miRDB Search Result.” [Online]. Available: https://mirdb.org/cgi-bin/search.cgi?searchType=miRNA&full=mirbase&searchBox=MIMAT0000689. [Accessed: 26-Feb-2023].
[175] “miRDB Search Result.” [Online]. Available: https://mirdb.org/cgi-bin/search.cgi?searchType=miRNA&full=mirbase&searchBox=MIMAT0004678. [Accessed: 26-Feb-

2023].
[176] J. S. Johansen *et al.*, “The potential diagnostic value of serum microRNA signature in patients with pancreatic cancer,” *Int. J. cancer*, vol. 139, no. 10, pp. 2312–2324, Nov. 2016.
[177] R. Liu *et al.*, “Serum microRNA expression profile as a biomarker in the diagnosis and prognosis of pancreatic cancer,” *Clin. Chem.*, vol. 58, no. 3, pp. 610–618, Mar. 2012.
[178] N. A. Schultz *et al.*, “MicroRNA biomarkers in whole blood for detection of pancreatic cancer,” *JAMA*, vol. 311, no. 4, pp. 392–404, Jan. 2014.
[179] X. Zhou, Z. Lu, T. Wang, Z. Huang, W. Zhu, and Y. Miao, “Plasma miRNAs in diagnosis and prognosis of pancreatic cancer: A miRNA expression analysis,” *Gene*, vol. 673, pp. 181–193, Oct. 2018.
[180] A. S, D. H, B. RE, and S. FH, “Differential Expression of MicroRNAs in Tissues and Plasma Co-exists as a Biomarker for Pancreatic Cancer,” *J. Cancer Sci. Ther.*, vol. 7, no. 11, 2015.
[181] S. Ali, K. Almhanna, W. Chen, P. A. Philip, and F. H. Sarkar, “Differentially expressed miRNAs in the plasma may provide a molecular signature for aggressive pancreatic cancer,” *Am. J. Transl. Res.*, vol. 3, no. 1, pp. 28–47, 2010.
[182] N. Gablo *et al.*, “Identification and Validation of Circulating Micrornas as Prognostic Biomarkers in Pancreatic Ductal Adenocarcinoma Patients Undergoing Surgical Resection,” *J. Clin. Med.*, vol. 9, no. 8, pp. 1–12, Aug. 2020.
[183] X. Kong *et al.*, “Detection of differentially expressed microRNAs in serum of pancreatic ductal adenocarcinoma patients: miR-196a could be a potential marker for poor prognosis,” *Dig. Dis. Sci.*, vol. 56, no. 2, pp. 602–609, Feb. 2011.
[184] F. van der Sijde *et al.*, “Serum miR-373-3p and miR-194-5p Are Associated with Early Tumor Progression during FOLFIRINOX Treatment in Pancreatic Cancer Patients: A Prospective Multicenter Study,” *Int. J. Mol. Sci.*, vol. 22, no. 20, Oct. 2021.
[185] P. Wang *et al.*, “The serum miR-21 level serves as a predictor for the chemosensitivity of advanced pancreatic cancer, and miR-21 expression confers chemoresistance by targeting FasL,” *Mol. Oncol.*, vol. 7, no. 3, pp. 334–345, 2013.
